# Mental health care for rare disease in the UK – recommendations from a quantitative survey and multi-stakeholder workshop

**DOI:** 10.1186/s12913-022-08060-9

**Published:** 2022-05-14

**Authors:** Rosa Spencer-Tansley, Nick Meade, Farhana Ali, Amy Simpson, Amy Hunter

**Affiliations:** Genetic Alliance UK, Creative Works, Blackhorse Lane, London, E17 6DS UK

**Keywords:** Rare disease, Undiagnosed, Mental health, Health service, Patient experience, Survey, Recommendations

## Abstract

**Background:**

Rare disease patients and carers report significant impacts on mental health but studies on UK populations have focussed on relatively few, specific conditions. Collectively rare conditions represent a substantial health burden, with an estimated 3.5 million affected individuals in the UK.

**Method:**

We explored the impact on mental health of living with a rare condition, and experiences of health service support, through an online survey. The survey assessed the impact of specific experiences commonly reported by those affected by a rare condition through multiple choice questions and Likert scale items, and open text question boxes. Through a multi-stakeholder workshop that involved facilitated discussion of our findings with patients/carers, clinicians and a government advisor, we developed recommendations for policy and practice toward a more person-centred and integrated approach.

**Results:**

Eligible responses came from 1231 patients and 564 carers. Due to their rare condition, the majority of respondents (> 90%) had felt worried/anxious; stressed; and /or low/depressed. Thirty-six percent of patients and 19% of carers had had suicidal thoughts.

Challenges that are particular to rare conditions and which negatively affect mental health included limited knowledge of the condition amongst healthcare professionals (88%), and not being believed or taken seriously by them.

Only 23% of respondents felt healthcare professionals considered mental and physical health as equally important. Almost half reported never having been asked about mental health by healthcare professionals. Our findings indicate that access to, and appropriateness of, professional psychological support needs to be improved. Peer group support is important but signposting is inadequate.

Our recommendations are for healthcare professionals to be supported to effectively and sensitively recognise and address patients’ and carers’ mental health needs; and for service level coordination of care to integrate professional psychological support with rare disease services.

**Conclusion:**

Living with a rare disease substantially impacts mental health. Many of the drivers of poor mental health reflect issues specific to managing rare conditions. To meet UK government commitments, there should be a focus on empowering healthcare professionals who treat rare disease patients and on integration of mental health support with rare disease services.

## Background

Although rare conditions by definition affect no more than 1 in 2000 individuals [1], there are over 6000 known conditions so that collectively they represent a substantial health burden, with an estimated 3.5 million affected individuals in the UK [2]. Studies of the psychological consequences of living with a rare condition for UK patients as a collective cohort are restricted to non-peer reviewed reports [3, 4]. Australian and American rare disease patient populations have been better studied and findings illustrate clear psychosocial and health-related quality of life impacts [5–9]. Recently a systematic review and meta-analysis of affective and anxiety disorders in adult patients with rare conditions reported prevalence estimates higher than the general population [10].

Further evidence is available from qualitative studies, which have generally indicated impaired emotional/psychological wellbeing for carers as well as patients. For example, psychological consequences have been reported amongst individuals, and parents/carers, living with undiagnosed or medically unexplained conditions [11, 12]; and parents/carers of individuals with specific diagnosed conditions [13–15].

Rare Disease UK’s survey of 1203 patients and carers reported numerous psychological consequences, yet the majority of respondents did not feel they received sufficient psychological support [4]. Similarly, of the 30 Australian families affected by rare disease that were surveyed by Anderson et al., the majority reported high levels of psychological stress but few had received psychological support [5].

Current literature does not provide a good understanding of the impact on mental health specifically in the UK, and in particular the impact of the support offered by the UK health services, for those affected by or caring for someone with any rare disease. The majority of the available evidence is condition-specific and/or is confined to validated tools that are time-restricted and do not provide information about patients’ and carers’ past experiences. We wanted to address this gap in knowledge for the UK rare disease population and investigate: the extent and nature of the impact on mental health; contributing factors; experiences of support and services in relation to mental health; and steps towards better mental health support. Secondly we aimed to use our findings to develop recommendations and facilitate the translation of policy into practice. The work is timely as there is a policy drive to improve mental health services in the UK, with sustained investment promised in order to ensure parity between physical and mental health [16–18]. Similarly, the UK health departments recently published an over-arching rare disease policy paper, the UK Rare Diseases Framework, which aims to inform the development of actions plans in England, Wales, Scotland and Northern Ireland [19]. The Framework commits to a person-centred approach for rare disease patients with an emphasis on aligning the implementation of the Framework with wider government policy on mental health and social care.

In this paper the term ‘carer’ is used to represent parents and other non-professional carers of individuals with rare conditions.

## Methods

**Fig. 1:**
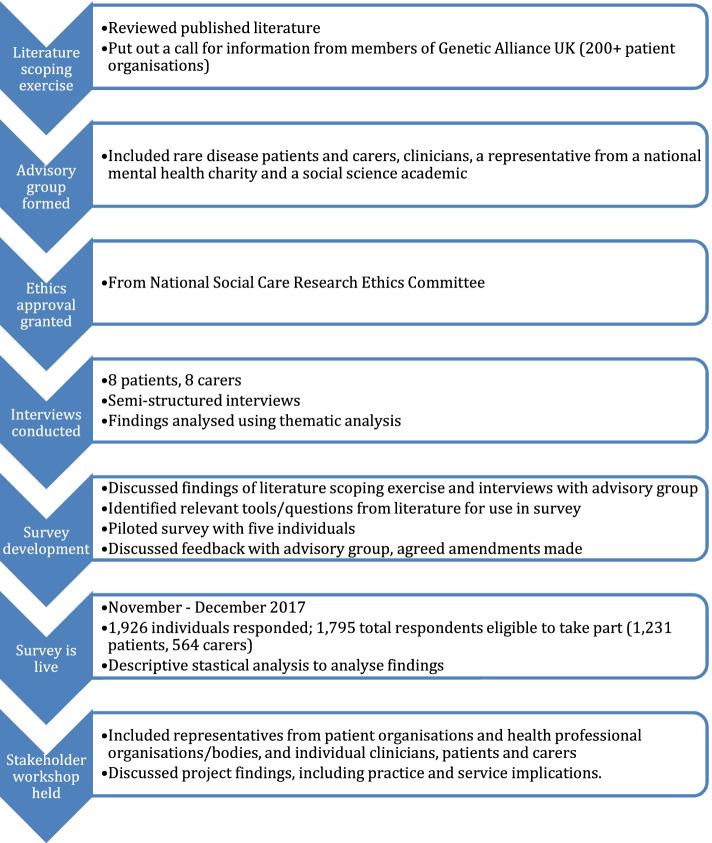
Flow chart of project methods

A schematic of the project method is shown in Fig. 1.

The elements in the survey were derived from: a review of relevant literature; a call for relevant information from members of Genetic Alliance UK (over 200 patient groups); and thematic analysis of transcripts from eight interviews with rare disease patients and eight interviews with carers about the impact of rare disease on mental health and their experiences of mental health support [20, 21]. An advisory group provided guidance on the survey development. The group included rare disease patients and carers, clinicians, a representative from a national mental health charity and a social science researcher. Data were handled in line with current data protection legislation. Descriptive statistics were generated from the data after cleaning to remove ineligible respondents and skipped questions, and the findings were used to create draft recommendations. The findings of the survey and interviews were discussed at a multi-stakeholder workshop in order to develop specific recommendations for change in policy and practice.

### Survey elements

The survey measured respondents’ self-assessments of their experience related to the following themes (for themselves or the person they care for): the emotional impact of rare disease; stressors and promoters of mental health; evaluation of care received; professional psychological support; other sources of support. Diagnostic information, whether the respondent was a patient or carer, the age of the person cared for, and respondent demographics were also collected.

The majority of the survey questions were quantitative and fixed-choice. Question types included single answers, multiple choice, and Likert scale items. A small number of questions included free-text responses.

The survey also included questions used in a previous study developed by The Neurological Alliance with their permission, an adaptation of the Short Warwick–Edinburgh Mental Well-being Scale (SWEMWBS), (© NHS Health Scotland, University of Warwick and University of Edinburgh), and items from the Multidimensional Scale of Perceived Social Support (MSPSS) [22–24]. The mixed-methods approach ensured the survey questions were relevant to the research question, and offered support for the survey findings through triangulation of results [25]. The survey was piloted with patients and carers and staff members of Genetic Alliance UK (five individuals in total). Volunteers piloted the survey in their own time and provided written or verbal feedback on comprehensiveness, sensitivity, relevance and ease of completion. Additional comments or suggestions were also encouraged. Amendments were made after discussion of the pilot with the advisory group.

### Recruitment

A link to the online survey was sent to members of Genetic Alliance UK; Rare Disease UK supporters (over 2000, including individuals and patient groups); and members of SWAN UK (syndromes without a name) which included more than 1800 families of children with an undiagnosed condition and more than 600 professionals. The online survey was also shared on social media to increase our reach to individuals beyond our immediate networks. The survey was hosted on SurveyMonkey and was live for a total of 28 days in November and December 2017.

Those eligible to take part were aged 18+, based in the UK, and either a patient with a rare disease, or a parent/carer. A set of screening questions ensured respondents met the eligibility criteria. Informed consent was obtained from all subjects via a consent box, which had to be ticked before the survey could be taken. Respondents had the option to withhold consent for the use of direct quotes from any free-text responses.

### Development of recommendations

Recommendations were drafted by the authors in response to the survey findings with the aim of improving services through empowering healthcare professionals and service-level coordination. The recommendations were refined through consultation at a multi-stakeholder workshop with 16 attendees including a psychologist, a behavioural therapist, representatives of rare disease patient organisations and mental health charities, individual patients and carers, representatives of professional bodies for healthcare professionals, academics, and a member of a government advisory committee.

The findings from the pre-survey interviews and from the survey were presented, followed by the draft recommendations, in order to stimulate constructive engagement. A whole-group discussion was facilitated to refine each recommendation to make sure they were achievable and specific. Notes of the discussion were taken by three note-takers. The notes were combined, then checked and refined collaboratively by the authors. The resulting text was marked up as relevant to policy and practice (relating to individual healthcare professionals or to service-level organisation), or out of scope. The recommendations were revised in light of the workshop report.

## Results

1. Respondent characteristics. Of the eligible respondents (1231 patients, 564 carers), completion rate was 69% for patients (913/1231) and 60% for carers (340/564). The skew toward female respondents is slightly greater for carers (92.4% female, 315/341) than for patients (83.9% female, 778/927). The high numbers of white British responses (94.8%, 1202/1268) is over-representative for the UK while other ethnicities were under-represented [26]. The four nations of the UK were represented in the sample approximately in proportion with population figures. The ages of respondents, and of the person looked after by respondents who are carers, are given in Tables 1 and 2.

**Table 1:** Age of survey respondents

Age	Respondents: patients n (%)	Respondents: carers n (%)	Total respondents: n (%)
18–24	61 (6.6)	0 (0)	61 (4.8)
25–40	265 (28.5)	144 (42.0)	409 (32.1)
41–60	452 (48.6)	174 (50.7)	626 (49.2)
61+	147 (15.8)	19 (5.5)	166 (13.0)
I’d prefer not to say	5 (0.5)	6 (1.7)	11 (0.9)
*Total*	930	343	1273

**Table 2:** Age of person cared for by respondents

Age of person cared for	Total n (%)
0–3	58 (16.9)
4 to 11	122 (35.6)
12 to 17	61 (17.8)
18+	94 (27.4)
I’d prefer not to say	8 (2.3)
*Total*	343

We presented an open text response box to collect as much diagnostic information as possible, and manually classified responses using the ERN (European Reference Networks) categories as a framework [27]. Most respondents fell under a single category. There was a relatively high proportion of respondents representing immunological, neurological, connective tissue, neuromuscular and endocrine conditions. Among the carers’ there was a high proportion of conditions categorised as congenital malformations and intellectual disability (including undiagnosed conditions with a suspected genetic origin, and chromosomal abnormalities). Some condition categories had little or no representation, including respiratory diseases, urogenital diseases, and paediatric cancer. Such a profile, while introducing a level of bias, is not unexpected given the dissemination method used.

### The nature of the emotional impact

#### Feelings and emotions

**Fig. 2:**
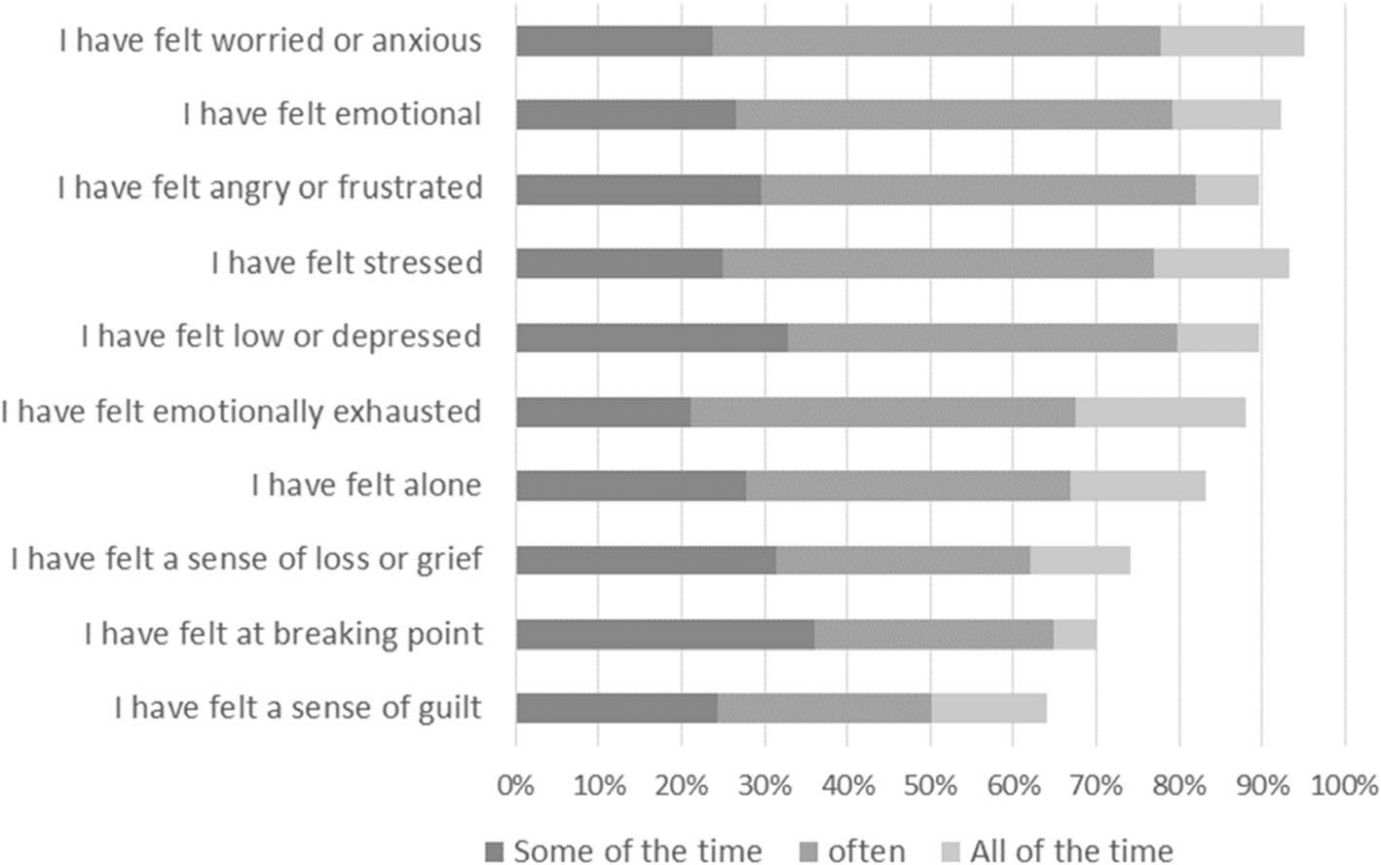
Emotions attributed to living with a rare condition (percent of respondents: patients and carers combined)

Respondents were given a list of thoughts and feelings and asked to rate each in terms of their experience of living with, or caring for someone with, a rare condition. Figure 2 illustrates the range of negative emotions that respondents reported. Almost all indicated that, as a result of the rare condition, they had felt (*some of the time/often/all of the time):* worried or anxious (95%, 1604/1688), stressed (93%, 1578/1689), emotional (92%, 1556/1685), low or depressed (90%, 1509/1683), angry or frustrated (90%, 1514/1690), emotionally exhausted (88%, 1485/1686), or alone (83%, 1403/1684). Thirty-six percent of patients (434/1194) and 19% of carers (92/496) had experienced thoughts about suicide (*some of the time/often/all of the time*).

Respondents had also experienced positive thoughts and feelings although this was less marked than for the negative feelings. The majority of respondents reported that, in relation to the rare condition, they had (*some of the time/often/all of the time):* been able to make up their mind (86%, 1455/1690), had been thinking clearly (77%, 1290/1683), had been dealing with problems well (77%, 1291/1688), feeling useful (62%, 1048/1679), close to others (60%, 1012/1684) and optimistic about the future (59%, 996/1687). Fewer than half of respondents (44%, 747/1685) reported feeling relaxed*;* the percentage was lower for carers (34%, 170/498) than for patients (49%, 577/1187).

#### Times that have had a negative impact on emotional health and wellbeing

The majority of survey respondents reported (*agree/strongly agree)* that the following periods of time had negatively impacted mental health: the day-to-day challenges of living with the condition (88%, 1367/1559), onset of symptoms (84%, 1290/1530), trying to get a diagnosis (83%, 1235/1490), coming to terms with the condition or diagnosis (83%, 1247/1506), thinking ahead to the future (82%, 1272/1558) and when a diagnosis was given (70%, 991/1418).

#### Knock-on impact of poor mental health

Respondents indicated that poor mental health had negatively impacted other aspects of life (*agree/strongly agree*). This included a knock-on impact on physical health (87%, 1242/1420), on work or studies (81%, 995/1222); and on personal relationships with partners (76%, 984/1292), friends (71%, 999/1414), and relatives (69%, 979/1411).

### Factors affecting mental health

The specific factors explored in relation to mental health can be grouped into three categories: i) interactions with healthcare professionals and services, ii) everyday living with a rare condition, and iii) additional factors for carers.

#### Interactions with healthcare professionals and services

**Fig. 3:**
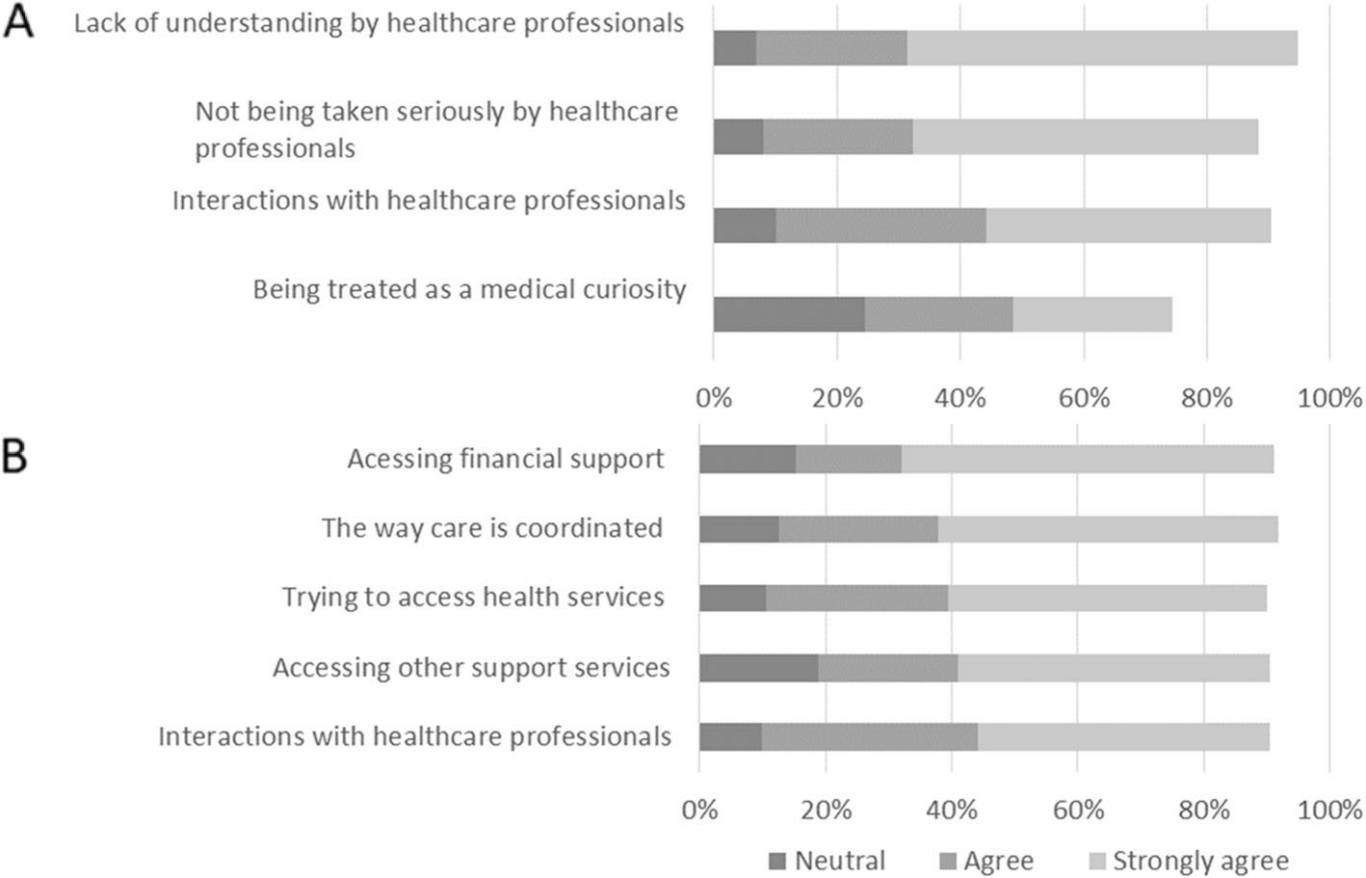
“The following things have had a negative impact on my emotional health and wellbeing”. Panel A shows the extent to which respondents agree that interactions with healthcare professionals have had a negative impact (percent of respondents, patients and carers combined). Panel B shows the extent to which respondents agree that interactions with services have had a negative impact (percent of respondents, patients and carers combined)

Respondents reported that aspects of their interactions with healthcare professionals had negatively impacted mental health (Fig. 3 panel A). Many of the factors identified were related to the rarity of the condition. For example, the most frequently identified factor *(agree/strongly agree)* was lack of awareness of the condition among healthcare professionals (88%, 1308/1486), followed by not being believed or taken seriously (80%, 1158/1444), and being treated as a medical curiosity (50%, 714/1433).

Factors relating to service access and coordination were also reported to negatively impact mental health (Fig. 3 panel B). This included (*agree/strongly agree*): trying to access health services or treatments (80%, 1183/1487), the way care is coordinated or organised between different departments or services (79%, 1161/1469), accessing financial support such as disability living allowance (76%, 951/1254), and accessing other support such as social care or respite care (72%, 945/1319).

#### Everyday living with a rare condition

Factors that are specific to everyday living with a rare condition were also reported to have impacted respondents’ mental health *(agree/strongly agree)*: lack of understanding about the condition among the public (90%, 1313/1458), feeling uncertain about what the future holds (87%, 1279/1469), having to explain the condition to other people (81%, 1191/1472), and lack of available information about the condition (76%, 1096/1448). Over half of respondents (56%, 792/1411) identified worrying information they had come across online as a factor affecting mental health.

For context we asked respondents about other stressors in their lives. A large majority of respondents reported (*agree/strongly agree)* that major life events (86%, 1196/1389), financial pressures and worries (80%, 1059/1327) and feeling socially isolated (76%, 1076/1412) had impacted mental health.

#### Additional factors for carers

Almost all carers reported that worrying about their child’s quality of life (97%, 370/383), and/or worrying about their child’s emotional wellbeing (96%, 365/381) had affected their own mental health *(agree/strongly agree).*

### Evaluation of care

The survey explored respondents’ experiences of services and how well they felt their emotional and mental health needs had been met.

#### Services, parity of esteem and information

**Fig. 4:**
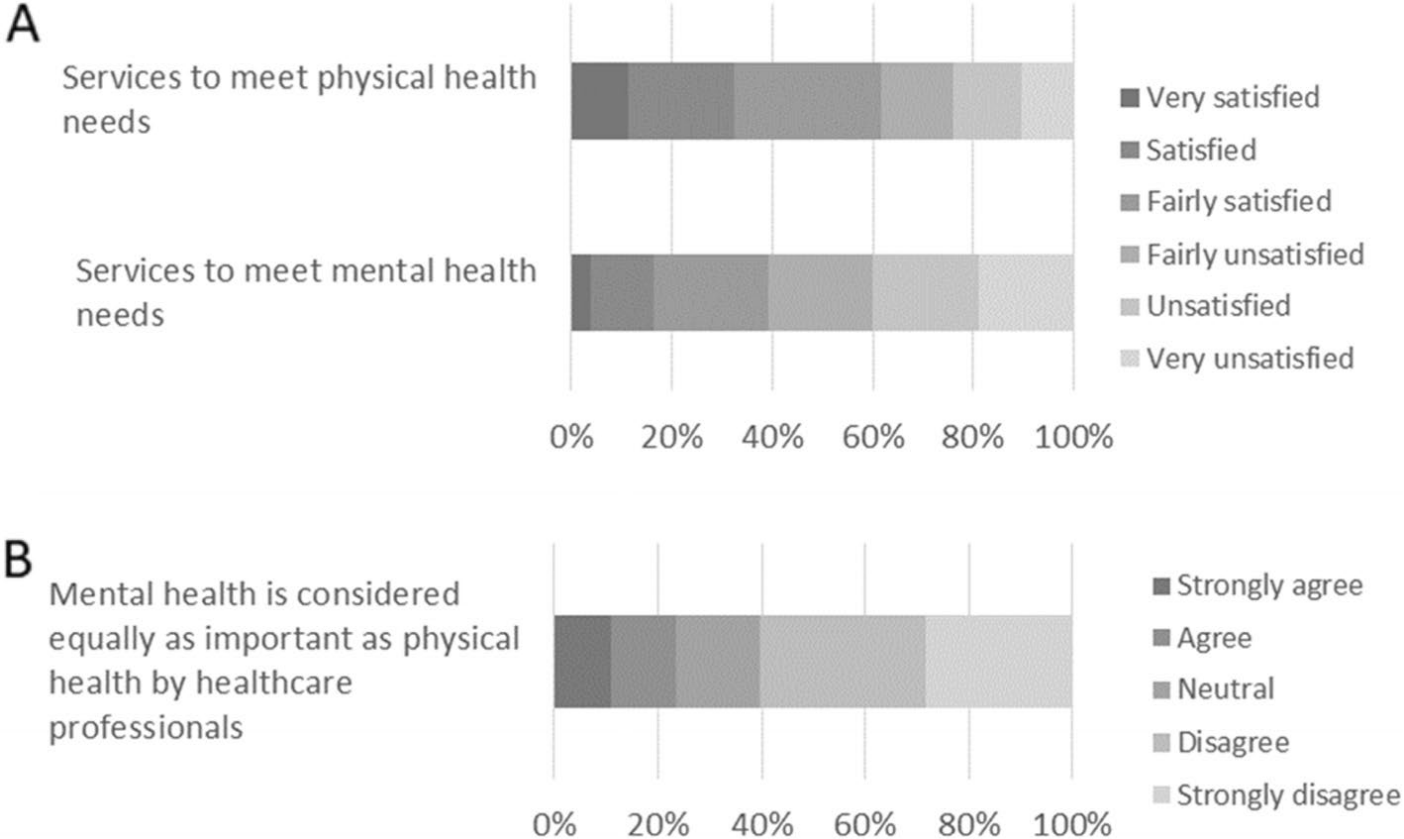
Evaluation of physical vs mental health services, and parity of esteem. Panel A shows respondents’ rating of physical vs mental health services. Panel B shows the level of agreement with physical and mental health having parity of esteem. Both panels show percent of respondents, patients and carers combined

Figure 4 panel A shows that, when rating the care and treatment they have received, 62% of respondents (852/1382) reported that they were satisfied *(fairly satisfied/satisfied/very satisfied*) with services to meet their physical health needs, but only 39% (532/1357) were satisfied with services to meet their mental health needs. In response to the statement “I feel my/my child’s mental health is considered equally as important as my/my child’s physical health by healthcare professionals involved in my/my child’s care,” the majority of respondents (60%, 814/1349) chose ‘*disagree’* or ‘*strongly disagree*’. A minority of respondents chose ‘*agree’* or *‘strongly agree’* (23%, 316/1349) (Fig. 4 panel B).

Approximately half of respondents were satisfied (*fairly satisfied/satisfied/very satisfied*) with the information they were given about the physical condition (48%, 653/1367), while fewer respondents (30%, 411/1366) were satisfied with the information provided about sources of emotional support.

#### Healthcare professionals asking about mental health

Respondents were presented with the statement “healthcare professionals ask about my/my child’s mental and emotional wellbeing’. Almost half of patients (46%, 454/988) and carers (48%, 173/362) chose *‘never’*, and just 8% of patients (81/988) and carers (28/362) chose *‘often’* or *‘always’*. Of carers, 57% (208/363) reported they had *‘never’* been asked about their *own* mental health by healthcare professionals involved in their child’s care.

**Fig. 5:**
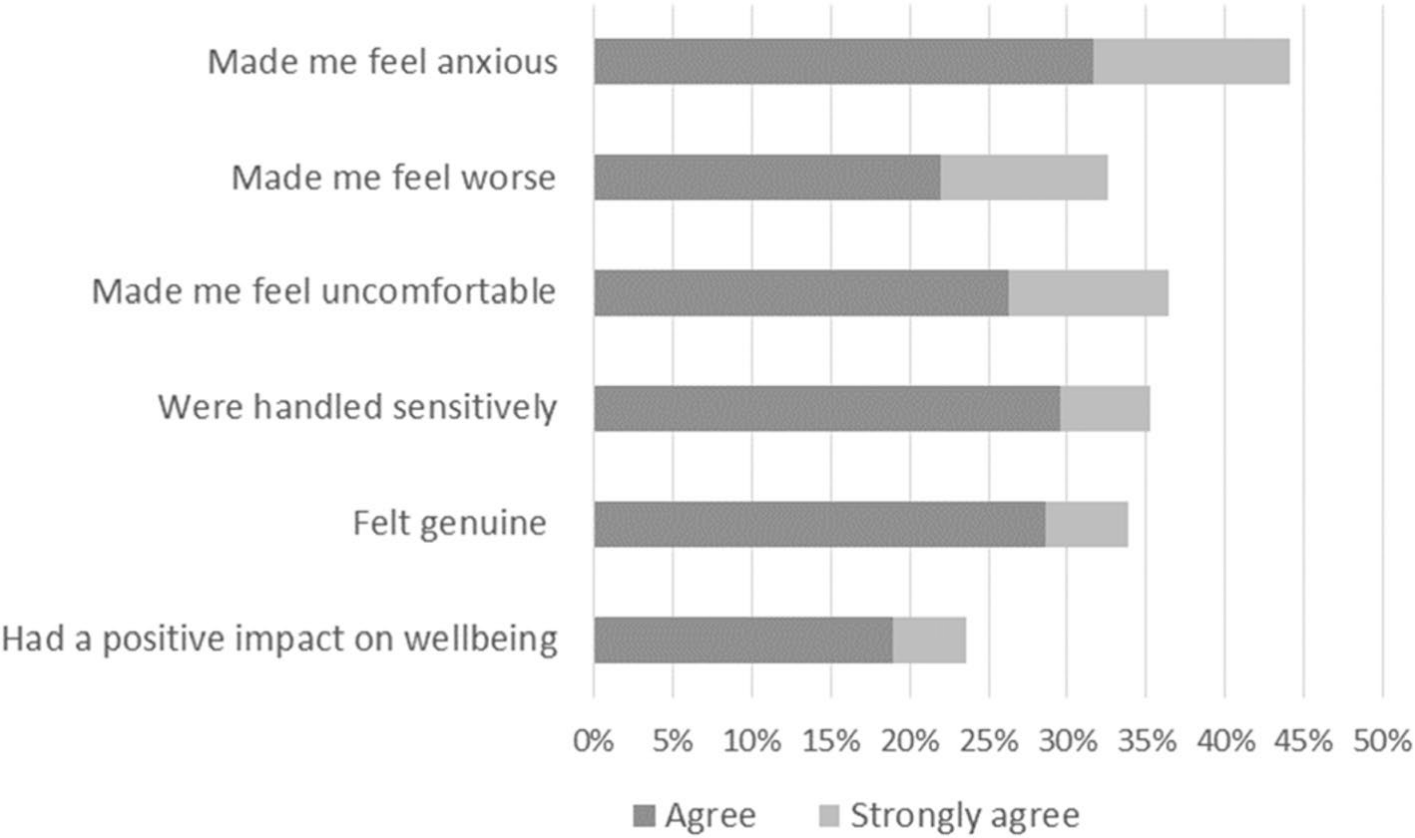
“Discussions about mental health and wellbeing with healthcare professionals … ”. Percent of respondents that agreed or strongly agreed with the statements; patients and carers combined

A proportion of respondents reported *(agree/strongly agree)* positive experiences when healthcare professionals had discussed mental health and wellbeing with them (Fig. 5): some reported that the discussions were handled sensitively (35%, 377/1068), that they felt genuine (34%, 369/1086) and that they had a positive impact on emotional wellbeing (24%, 252/1066). Conversely respondents also reported *(agree/strongly agree)* negative experiences when discussing mental health and wellbeing with healthcare professionals (Fig. 5): discussions made them feel anxious (44%, 468/1061); uncomfortable (36%, 386/1060) or made them feel worse (34%, 356/1059).

#### How to improve care to better support wellbeing and mental health

A large majority of respondents felt the following would improve their mental health *(agree/strongly agree)*: greater awareness of the emotional challenges of living with a rare condition among healthcare professionals (91%, 1184/1308), greater sensitivity among healthcare professionals (85%, 1090/1284), and being asked more frequently about mental health and wellbeing by healthcare professionals (81%, 1056/1296). Respondents also reported that easier access to emotional support would improve their mental health; the majority felt *(agree/strongly agree*) that easier access to professional psychological support (85%, 1069/1262) and better signposting to alternative sources of emotional support (86%, 1105/1283), would benefit their mental health.

### Experiences of professional psychological support

#### Access and barriers to professional psychological support services

Just over half (54%,719/1323) of respondents had not accessed any professional psychological support. Of those who had accessed professional psychological support just 7% had accessed it through a specialist clinic for their condition (41/588) and only 2% of respondents (14/588) had been offered professional psychological support at the time of diagnosis of their condition. More respondents had been referred by their GP (48%, 280/588) or clinicians at their hospital (21%, 123/588). A proportion of respondents (18%, 106/588) had accessed private psychological support (i.e. ‘arranged and paid for it myself’). Of these respondents, over half (55%, 58/105) had paid more than £500 in total and a further quarter (26%, 27/105) had paid between £100 and £500.

Respondents identified several factors that had prevented them from accessing professional psychological support. The most commonly reported was that it had not been suggested by healthcare professionals (41%, 527/1285), followed by not being able to afford private psychological support (29%, 372/1285) and that the waiting lists for mental health services were too long (23%, 299/1285). Carers in particular cited too much pressure on their time as being a barrier (22%, 77/350).

#### Evaluation of professional psychological support

**Fig. 6:**
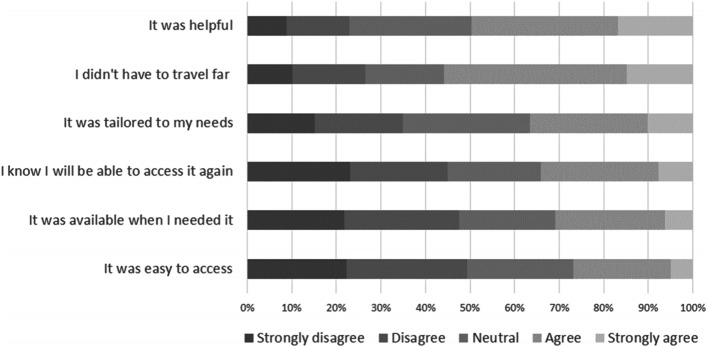
Evaluation of professional psychological support received. Extent to which respondents agreed with statements about the support received. Percent of respondents, patients and carers combined

Respondents who had accessed professional psychological support were asked to evaluate that support (Fig. 6. Around half (49%, 282/570) had found it was helpful *(agree/strongly agree)*, but fewer (37%, 209/571) felt it was tailored to their needs *(agree/strongly agree).*

Over half of respondents (56%, 319/571) reported (*agree/strongly agree*) they did not have to travel far to access the support. Fewer felt that the support was available when needed (31%, 179/578) or had been easy to access (27%, 155/580) or were confident they would be able to access professional psychological support again if they needed it (34%, 198/580) (*agree/strongly agree*).

### Support from other sources

**Fig. 7:**
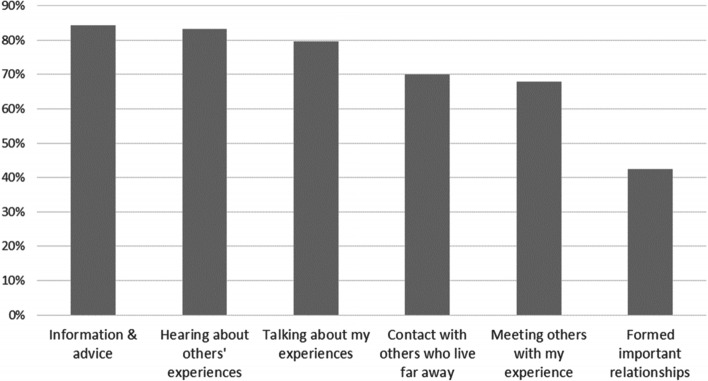
Ways in which other sources of support are helpful. Percent of respondents who agreed or strongly agreed with answers to the question “How has this support been helpful to you?”. Patients and carers combined

Fifty-nine percent of respondents (759/1292) reported they had accessed additional emotional support such as peer support online (81%, 617/759) or face-to-face (36%, 274/759) and support from charities or the community sector (25%, 192/759). The majority of respondents (75%, 534/713) who had accessed such support reported having found it themselves. A minority had been signposted to the support by a healthcare professional (18%, 125/713), and the most frequent reason for not accessing such support was not knowing how to access it (41%, 210/516). Eighteen percent of respondents believed there was no such support available to them (92/516). Respondents indicated (*agree/strongly agree)* that such sources of support were helpful for several different reasons (Fig. 7).

Over half of respondents (67%, 862/1282) reported that their family ‘really tries to help them’ (*agree/strongly agree*) but fewer felt that they get the emotional support they need from their family (48%, 612/1281). The equivalent numbers for friends were: 47% (588/1256) and 37% (462/1257).

## Discussion

### The nature of the emotional impact of rare disease

The current study is significant in that it is not restricted to a specific rare disease type and has a broad scope of investigation into the UK population of people affected by rare conditions, including the nature of the impact of living with a rare disease, what factors affect mental health, experiences of support and services, and what patients and carers judge would improve mental health support. Our findings demonstrate that rare disease has a substantial negative impact on mental health for both patients and carers. This reflects existing literature on unmet needs [3, 5, 28] and is consistent with findings from condition-specific studies which used diagnostic tools to measure anxiety and/or depression in rare disease patients and/or carers and impact on quality of life [8, 10, 29–32].

Consistent with condition-specific research [32–35], our findings show that the negative impact on mental health can be chronic, and can occur or recur at different times such as during the ‘diagnostic odyssey’; when a diagnosis is given; and while coming to terms with the condition. This highlights the periods when additional emotional support may be particularly important. The impact of the ‘diagnostic odyssey’ is concerning: it can last a long time, with one in four waiting longer than five years for a diagnosis [4] and many will not see the end of this phase, as the diagnostic yield of genome sequencing remains significantly lower than 100% [36]. This means that the mental health of rare disease patients is affected before they are categorically defined as such, and before they might access support through specialist services or disease-specific patient support groups. Other groups have asserted that receiving a rare disease diagnosis can be a particularly emotionally challenging time, in which patients and families experience feelings such as shock, meaning routine psychological support should be offered at the time of diagnosis [5]. It may seem counterintuitive that both not having a diagnosis, and receiving a diagnosis, can lead to poor mental health, but both offer challenges; for example, the anxiety of not knowing what is ‘wrong’ on one hand, and the shock of a poor prognosis on the other. Our study indicates that patients and carers need ongoing support in addressing the emotional challenges that a rare condition can present. Future research should focus on individuals’ experiences during these periods, and use validated tools to assess interventions.

While almost all of our survey respondents reported negative emotions in relation to their condition, many also reported positive thoughts and feelings, and they described a variety of coping mechanisms during an earlier qualitative study [20, 21]. It would be valuable to explore further what factors promote positive adjustment and coping in people living with rare diseases and any differences to those living with common chronic conditions.

### Factors affecting mental health

An important finding from this study is that many of the drivers of poor mental health are associated with the challenges of managing a condition that is rare. This includes a lack of understanding about the condition among the public, feeling uncertain about what the future holds, and having to explain the condition to other people. Similarly, many contributing factors arising from interactions with health and other services reflected issues associated with the rarity of the condition. For example, a lack of awareness among healthcare professionals, not being believed or taken seriously, and being treated as a medical curiosity. Factors related to service access were also likely related to the rarity of the condition, in particular the way care is coordinated; it is well established that care is often poorly coordinated for rare disease patients, many of whom have complex needs and are under the care of several doctors from different specialties [4].

Our findings are consistent with previous condition- and country-specific research into the psychological impact of the process of diagnosis [33–35], and the experiences of Australian families affected by rare diseases that found that delay in diagnosis and the perceived lack of knowledge among healthcare professionals were associated with anxiety and stress [7]. This study also reported that parents perceived the lack of knowledge among clinicians to be a leading cause of diagnostic delays, highlighting an interrelation between two emotional stressors. Similarly, interviews conducted with caregivers of children with inherited metabolic conditions [14] identified emotional challenges associated with poor coordination of healthcare services and there is growing evidence about the burden of time and energy required to manage numerous appointments across different specialisms [37, 38]. Having symptoms questioned by healthcare professionals, or being mislabelled with a psychological health issue when trying to seek help for physical symptoms, have also been reported as triggers of poor mental health in specific patient populations [11].

It is known that long-term conditions significantly increase the risk of mental health issues [39, 40]. Such associations have been the subject of policy-level initiatives in the UK, such as the ‘Five Year Forward View on Mental Health’ report which emphasised the need to address the link between physical and mental health [17]. The interrelation between physical and mental health is complex and poor mental health can cause physical deterioration [41, 42]. Our findings indicate that those affected by rare conditions may face additional stressors because of the rarity of their condition. As such they may be particularly vulnerable to experiencing mental health issues.

Patients do not live in a vacuum: our survey highlights that patients and carers face the challenges of everyday life such as financial pressures, major life events, social isolation, and employment issues along with those directly associated with their condition. It may be that such factors are exacerbated by the rarity of their conditions. For example, social isolation is likely to be a particularly pertinent issue for rare disease patients and carers due to factors such as poor understanding of the condition among the public and peers; while previous research has highlighted the considerable financial costs associated with managing rare conditions that patients and carers face [4, 43].

### Practice implications based on our findings

The recommendations arising from this study, around the empowerment of healthcare professionals and service-level coordination, are targeted at specific government departments, advisory bodies, professional medical bodies and medical schools [20]. They are consistent with a recent international scoping review focussed on the emotional impact of the diagnostic process for rare disease which proposes improvements at both the service and individual healthcare provider level [44]. Alongside other patient organisations, healthcare professionals and NHS policy makers, Genetic Alliance UK contributed to the new UK Rare Diseases Framework which includes the aim of aligning rare disease care with wider policy development such as mental health support [19].

### Recommendations for the empowerment of healthcare professionals

1. Healthcare professionals should be provided with the skills, knowledge and capacity to:
  demonstrate awareness of the challenges of living with a rare disease,
  handle discussions about mental health sensitively.
2. Healthcare professionals should routinely signpost patients and carers to sources of support.

Molster et al. advocated for greater awareness of rare diseases among clinicians, to improve the diagnostic process and ensure informational needs are met [6]. We propose that this should also extend to greater awareness of the emotional challenges of rare disease. Healthcare professionals could show greater sensitivity when delivering a diagnosis to patients and carers, for example [7]. Our findings also indicate a need for greater sensitivity when interacting with patients without a diagnosis. This is consistent with previous studies exploring the experiences of patients living without a diagnosis [11, 12, 37].

Our respondents were clear that they would benefit from healthcare professionals asking more frequently about mental health, with the majority reporting that they are never or infrequently asked about mental health. To our knowledge this is the first study to assess the frequency of such conversations, although the finding is reflected in a report that suggests patients with rare neurological conditions are less likely to be asked about their mental health than neurology patients with more common conditions [45]. Given that many rare disease patients have frequent contact with healthcare professionals, this is alarming [4]. It is vital that healthcare professionals are provided with training so they are aware of the emotional challenges faced by their rare disease patients, they are able to communicate with sensitivity, and are equipped to handle discussions around mental health. This is underscored by our finding that while some respondents had positive experiences when discussing mental health with healthcare professionals, for others the conversations had directly caused increased levels of anxiety.

It may be that healthcare professionals would feel more comfortable raising the subject of mental health if they know that appropriate professional psychological support is available. Healthcare professionals need to be confident about how to make such referrals, but the current level of mental health service provision might be a barrier as cuts to funding of psychological support services have resulted in high thresholds for access and long waiting times [46].

Additional sources of emotional support, such as peer support and patient organisations, have been shown to be important to rare disease patients and carers; our findings support this and add detail about why it is valued [4, 5, 7]. However, in line with other studies, fewer than half of our respondents had accessed such support [4, 5]. Our survey indicates this is largely because of not knowing how to find it and that healthcare professionals are not routinely signposting to additional sources of support, possibly due to a lack of knowledge [3]. Training and professional development should therefore incorporate information about signposting.

There are resources available to help healthcare professionals signpost to support in the UK, for example on the website of Genetic Alliance UK [47]. Organisations do not exist for every rare condition, but there is a growing number of online communities (using platforms such as Facebook and Rare Connect) connecting individuals across geographical areas [48]. Online support was by far the most frequently utilised of all additional sources of support among our survey respondents.

We found that patients and carers face multiple barriers to accessing professional psychological support, such as it not being suggested by healthcare professionals, and a large majority of our respondents also felt that easier access to professional psychological support would improve their mental health. Our study echoes findings from research in Australia, the UK and across Europe [4, 6, 28]. Our findings also indicate that when professional psychological support is accessed it could be more effective and better tailored to patients’ needs. This is consistent with a survey of UK-based individuals with neurological conditions [45].

### Recommendation for service-level coordination

3. Coordinated rare disease services should include assessment of mental health needs and access to mental health services. This should be extended to carers.

Parity of esteem is a principle in which mental health is given equal priority with physical health. Our data indicate problems with access to effective professional psychological support, and a lack of parity of esteem between physical and mental health. The gold standard for rare disease care is generally held to be coordination through multidisciplinary team working and previous studies in Australia have recommended that psychological support is “embedded in RD services” and is routinely available following diagnosis [5, 7]. We propose that assessment of mental health and a care pathway to support services, as part of coordinated care, be instigated for rare disease patients and carers in the UK. Evidence of the positive impact of psychological support for carers of people with rare diseases is growing [49].

Very few of our respondents received psychological support via specialist rare disease centres. NHS England and the UK government have made several commitments to make mental health a key healthcare focus, with promises of sustained investment, in order to ensure parity between physical and mental health [17, 18]. The new UK Rare Diseases Framework flags the need for mental healthcare to be planned alongside physical healthcare [19]. Our recommendation aligns with these national commitments and underscores the need for them to be implemented.

There is a similar need for national plans and mainstream policies to improve mental health support for rare disease patients and their carers in other nations. While 25 of the EU member states have national plans, a commitment to improved psychological support is still lacking [50]. National policy in North America, Asia and Australia is largely focussed on research and treatment innovations, although the Canadian Organization for Rare Disorders has developed a national strategy and is calling for its implementation [51, 52]. Change in all regions may be encouraged by the recognition of rare diseases by the World Health Organization as a priority disease area, and its identification of psychosocial care as an area of unmet need [53].

The value of undertaking research into patients’ and carers’ experiences across rare diseases as a collective, in a healthcare system that is oriented towards more common diseases, has been highlighted by previous researchers, as it will “raise the profile of rare diseases and is likely to result in less duplication of efforts and resources across the range of rare diseases.” [6].

We did not set out to address the question of whether there is under-diagnosis of mental health conditions amongst rare disease patients and carers and our study does not allow us to report outcomes equivalent to a standardised screening approach, or make comparisons with other populations. However our approach has allowed for a broader understanding of patients’ and carers’ experiences of their mental health now and historically (this would not be generated by standardised tools which measure outcomes based on a very short time window). An additional strength is the inclusion of stakeholders throughout the project, on the project advisory group and through the multi-stakeholder workshop.

Our sample was self-selecting therefore it is not possible to determine the generalisability of our findings. There could be a bias toward those with more difficult experiences of mental health. Additionally, the sample was strongly biased toward women, which may be because women are more likely to respond to research requests of this nature and are more likely to take on the primary care role [28].

Further consultation with patients, carers and service providers, with experience of different types of rare disease, is needed to tailor guidelines for implementation of our recommendations. We expect that our recommendations, through feeding into national policy work, will provide the foundation for this progress to be made.

## Conclusions

To our knowledge this is the largest UK-based study to systematically explore the impact of living with a rare disease on mental health. Our findings indicate a substantial impact on mental health for both patients and carers and that many drivers of poor mental health reflect issues that are specific to managing a condition that is rare. Based on our findings and discussion with stakeholders we recommend that healthcare professionals should be provided with the skills, knowledge and capacity to demonstrate an awareness of the challenges of living with a rare disease; and that healthcare professionals should routinely signpost to sources of support. We also recommend that coordinated rare disease services should include assessment of mental health needs and access to mental health services, and that this should be extended to carers. If effectively implemented, these changes could do much to address some of the mental health issues patients and carers in the UK currently face; and to ensure that mental health is considered as important as their physical health. Since our data were analysed, the covid-19 pandemic has been a significant additional stressor for the mental health and wellbeing of rare disease patients and carers, making improvements in support at a national level even more urgent [54].

## Data Availability

The full survey is available on request.

## Abbreviation

ERN: European Reference Networks
